# Evaluation of Up-Converting Phosphor Technology-Based Lateral Flow Strips for Rapid Detection of *Bacillus anthracis* Spore, *Brucella* spp., and *Yersinia pestis*

**DOI:** 10.1371/journal.pone.0105305

**Published:** 2014-08-21

**Authors:** Pingping Zhang, Xiao Liu, Chengbin Wang, Yong Zhao, Fei Hua, Chunfeng Li, Ruifu Yang, Lei Zhou

**Affiliations:** 1 Laboratory of Analytical Microbiology, State Key Laboratory of Pathogen and Biosecurity, Beijing Institute of Microbiology and Epidemiology, Beijing, People's Republic of China; 2 School of Public Health and Management, Chongqing Medical University, Chongqing, People's Republic of China; 3 Department of Clinical Laboratory, Chinese People's Liberation Army General Hospital, Beijing, People's Republic of China; 4 Department of Etiology, Taishan Medical University, Tai'an, People's Republic of China; INIAV, I.P.- National Institute of Agriculture and Veterinary Research, Portugal

## Abstract

*Bacillus anthracis*, *Brucella* spp., and *Yersinia pestis* are zoonotic pathogens and biowarfare- or bioterrorism-associated agents that must be detected rapidly on-site from various samples (e.g., viscera and powders). An up-converting phosphor technology-based lateral flow (UPT–LF) strip was developed as a point-of-care testing (POCT) to satisfy the requirements of first-level emergency response. We developed UPT–LF POCT to quantitatively detect the three pathogens within 15 min. Sample and operation-error tolerances of the assay were comprehensively evaluated. The sensitivity of UPT–LF assay to bacterial detection reached 10^4^ cfu·mL^−1^ (100 cfu/test), with a linear quantitative range of 4 to 6 orders of magnitude. Results revealed that the UPT–LF assay exhibited a high specificity with the absence of false-positive results even at 10^9^ cfu·mL^−1^ of non-specific bacterial contamination. The assay could tolerate samples with a wide pH range (2 to 12), high ion strengths (≥4 mol·L^−1^ of NaCl), high viscosities (≤25 mg·mL^−1^ of PEG20000 or ≥20% of glycerol), and high concentrations of bio-macromolecule (≤200 mg·mL^−1^ of bovine serum albumin or ≥80 mg·mL^−1^ of casein). The influence of various types of powders and viscera (fresh and decomposed) on the performance of UPT–LF assay was determined. The operational error of liquid measurement exhibited few effects on sensitivity and specificity. The developed UPT–LF POCT assay is applicable under field conditions with excellent tolerance to sample complexity and operational error.

## Introduction

Anthrax, brucellosis, and plague, which are caused by *Bacillus anthracis*, *Brucella* spp., and *Yersinia pestis*, respectively, are infectious zoonotic diseases with high mortality rates. These diseases have persisted in several countries with a wide range of animal hosts, susceptible animals, vectors, or soils as reservoirs in nature foci [1]–[3]. Moreover, anthrax, brucellosis, and plague have become threats to public health because their corresponding pathogens are easily transmitted from contaminated animal products or environment materials to humans through gastrointestinal, respiratory, and integumentary systems [1]–[4]. The pathogens have been used in biological warfare (biowarfare) and biological terrorism (bioterrorism) because of their wide distribution, simple culture needs, and aerosol dissemination capability [2], [5]–[7]. Pathogen spread by human-to-human respiratory transmission has worsened the problem [2], [8].

Point-of-care testing (POCT) assays are necessary for surveillance in nature foci and first-level emergency responses in biowarfare and bioterrorism through on-site detection of the pathogens (e.g., viscera and decomposed viscera, blood, excretion, soil, water, and food). The simplicity and error tolerance of detection methods should also be considered for nonprofessional operations. Polymerase chain reaction (PCR) [4] and other techniques [9]–[13] have been developed, but these methods cannot be used as POCT assays because of expensive equipment, complex sample pretreatment, and complicated and time-consuming operations. Lateral flow (LF) immunoassay is a widely used and comprehensively applied POCT technology [14], [15] to rapidly detect various pathogens, such as *Y. pestis* [16], *B. anthracis* [17], and *Brucella* spp. [18]. However, traditional LF assays that use colloidal gold as label have the following limitations under potential bioterrorism scenarios with mixed bacteria and complex samples: (i) absence of reactions with different strains of the same pathogen species, (ii) low sensitivities caused by macroscopic observations, (iii) results cannot be analyzed because of serious interferences by colored samples such as whole blood [16], and (iv) inapplicability in extreme conditions (e.g., acid, basic, saline, viscose, and protein-rich solutions) because of the interference in conjugation between antibodies and gold particles that depend on physical adsorption. To simultaneously detect different strains of the same pathogen species, specific antigens are used to prepare antibodies, and two or more antibodies are mixed in LF assays to efficiently recognize species of *B. anthracis* [19], *Brucella* [20], [21], and *Y. pestis* [16], [22] under field conditions. However, only a few works have focused on the rapid detection of pathogens from complex samples using LF assay to meet the requirements of POCT diagnostic and system assessments using extreme reagents and on-site samples. The rarity of studies on LF assay-based pathogen detection is attributed to the limitation of colloidal gold as a biolabel.

An up-converting phosphor technology-based lateral flow (UPT–LF) assay was developed with at least 10-fold improvement using a nanometer-sized luminous up-converting phosphor (UCP) particle as the biolabel [23]. Given the unique optical characteristics of UCP and the covalent bond between UCP and biomolecules, UPT–LF assay exhibits robust performance to detect blood [24]–[26] and saliva [27] samples. UPT–LF assays have also been successfully used to quantitatively detect bacteria [28]–[30], viruses [31], biomolecules [25], [32], and drugs of abuse [28]. The assays can potentially be applied to POCT diagnostics upon improvement because of their less background noise, high sensitivity, low-cost operation, and stable photochemical properties [26], [33].

This study aims to improve the UPT–LF assay for point-of-care applications. The assay performance was comprehensively evaluated to rapidly detect *B. anthracis*, *Brucella* spp., and *Y. pestis* under the interference of various factors. With this method, surveillance in nature foci and first-level emergency response in bioterrorism can be performed.

## Materials and Methods

### Ethic statement

Eight-week-old female Balb/c mice were obtained from the Laboratory Animal Research Center, Academy of Military Medical Sciences (China). Mice acquisition was given license by the Ministry of Health in the General Logistics Department of Chinese People's Liberation Army (Permit No. SCXK-2007-004). All experiments were approved by the Committee of the Welfare and Ethics of Laboratory Animals, Beijing Institute of Microbiology and Epidemiology (Beijing, China). All mice were housed in an accredited research animal facility fully staffed with trained personnel. Humane endpoints were strictly observed. Balb/c mice were humanely culled by cervical dislocation.

### Reagents and materials

UCP (NaYF_4_:Yb^3+^,Er^3+^) with a diameter of approximately 50 nm was prepared and provided by Dr. Yan Zheng from Shanghai Kerune Phosphor Technology Co., Ltd. (Shanghai, China). The excitation and emission spectrum peaks of UCP were 980 nm and 541.5 nm, respectively.

Nitrocellulose membrane (SHF 1350225) and glass fiber (GFCP20300) were obtained from Millipore Corp. (Bedford, MA, USA). Papers (Nos. 470 and 903) were purchased from Schleicher & Schuell, Inc. (Keene, NH, USA). Plastic cartridges and laminating cards were manufactured by Shenzhen Jincanhua Industry Co. (Shenzhen, China) and Shanghai Liangxin Biotechnology Co. (Shanghai, China), respectively; both materials were designed by our group.

Agar powder, bovine serum albumin V (BSA), casein, tryptone, yeast extract powder, Ca(NO_3_)_2_, HCl, FeSO_4_, glycerin, MgSO_4_, MnCl_2_, PEG20000, KCl, NaN_3_, NaCl, and NaOH were all purchased from Sigma–Aldrich (St. Louis, MO, USA). All other reagents were of analytical grade, used without further purification, and supplied by Sinopharm Chemical Reagent Co., Ltd. (Shanghai, China) unless otherwise specified. Acid instant drink, alkaline putty powder, flour, gourmet powder, milk powder, and sugar were purchased from a local supermarket. Viscera of Balb/c mouse, including heart, liver, lung and spleen, were homogenized and divided into two parts. One part was stored at −20°C as fresh specimens, and the other was incubated at 37°C for two weeks as decomposed specimens.

### Bacterial cultures and monoclonal antibody preparation

*B. anthracis*, *B. melitensis* M55009, and *Y. pestis* were used for specific detection. The following bacteria were used to evaluate the specificity: *Bacillus atrophaeus* (two isolates), *B. cereus* (three isolates), *B. subtilis* (three isolates), *B. thuringiensis* (two isolates), *Escherichia coli* O157:H7, *Salmonella choleraesuis*, *S. enteritidis*, *S. paratyphi* A, *S. paratyphi* B, *S. paratyphi* C, *S. typhi*, *S. typhimurium*, *Vibrio cholerae* O1, *V. cholerae* O139, *Y. aldovae*, *Y. enterocolitica*, *Y. intermedia*, *Y. kristensenii*, *Y. mollaretii*, *Y. pseudotuberculosis*, *Y. rohdei*, and *Y. ruckeri*.

To prepare the spores, *B. anthracis*, *B. atrophaeus* (two isolates), *B. cereus* (three isolates), *B. subtilis* (three isolates), and *B. thuringiensis* (two isolates) were grown on nutrient agar plates at 37°C for 7 d. The plates contained 6 g·L^−1^ tryptone, 3 g·L^−1^ yeast extract powder, 10 g·L^−1^ NaCl, 1 g·L^−1^ KCl, 0.122 g·L^−1^ MgSO_4_, 0.23 g·L^−1^ Ca(NO_3_)_2_, 0.197 g·L^−1^ MnCl_2_, 0.0002 g·L^−1^ FeSO_4_, and 15 g·L^−1^ agar powder. Vegetative cells were placed in sterilized distilled water and lysed by osmotic pressure. The spores were collected by scraping the cells into sterilized saline and pelleted at 7,000 rpm for 15 min. The pellets were washed thrice through resuspension and centrifugation in sterilized saline. The resultant spores exhibited 99% purity through microscopic observation; spore population was determined by Luria–Bertani (LB) agar plate count. *V. cholerae* and other bacteria were cultured in alkaline peptone water and LB broth at 37°C, respectively. Pure cultures were collected at the logarithmic phase through centrifugation and resuspension in sterilized saline. The population was determined by LB plate count.

To prepare monoclonal antibodies (mAb), eight-week-old female Balb/c mice were immunized every two weeks for two months by subcutaneous injection. To prepare mAb against *Y. pestis*, each mouse was injected with 25 µg F1 antigens [30]. Subsequently, 2.5×10^8^ cfu of Sterne strain, a vaccine strain of *B. anthracis*, was inactivated by 4% formaldehyde solution for immunization. Several mAbs were obtained following preliminary preparation. The antibodies were then screened by ELISA coated by Sterne and 170044 stains. The mAbs positive for the two strains in the ELISA assay were chosen [34]. To prepare mAbs against *Brucella*, 2.5×10^8^ cfu of three strains, namely, *B. abortus* S19, *B. suis* S1, and *B. melitensis* M55009 strains, was mixed and inactivated for immunization. Then, preliminary mAbs were screened by ELISA assay coated by the three strains [29].

### Establishment of UPT–LF assay

**Figure 1:**
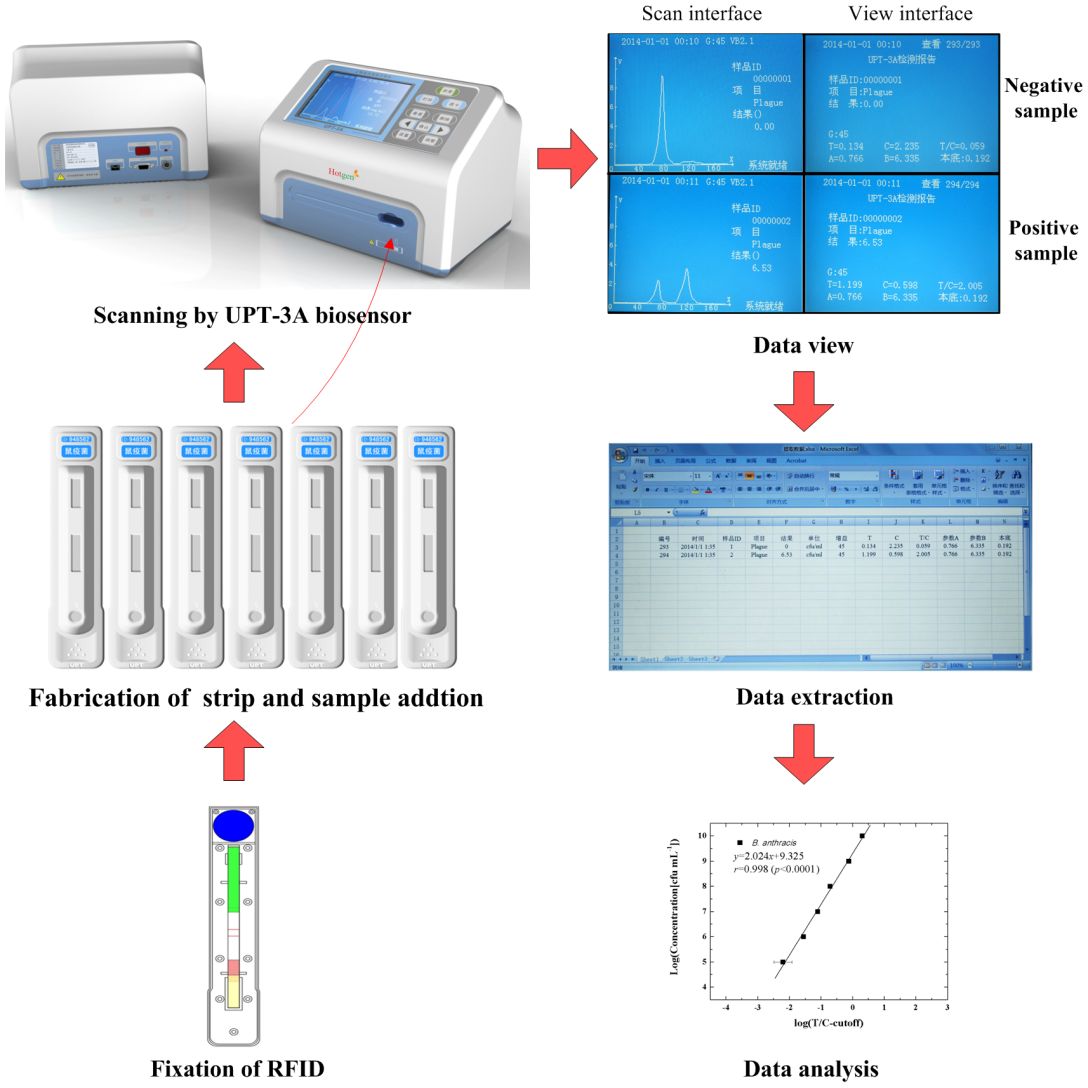
Workflow for pathogen detection using the UPT–LF biosensor. UPT–LF strips fixed with RFID are fabricated first. The samples are added to each strip every 30 s. Afterwards, the strips are scanned by an upgraded UPT-3A biosensor. The dimension of the upgraded UPT biosensor is 32 cm×21 cm×20 cm. The power sockets, powder switch, and USB interface are installed on the rear panel (left), and the integrated keys are installed on the front panel (right). When the “measure” key (marked in Chinese) at the top of panel is pressed, the scanning image is shown in the window. After the “view” key is pressed, the results are shown in detail. The data are exported for analysis using Excel through the USB interface to perform data analysis.

UPT–LF strips were prepared as previously described [29]–[31] to detect *B. anthracis*, *Brucella spp.*, and *Y. pestis*, and the strips were named Ban-UPT–LF, Bru-UPT–LF, and Ype-UPT–LF, respectively. The mAb coating corresponding to each bacterium (1.0 mg/mL) and goat anti-mouse IgG (2 mg/mL) was dispensed on a nitrocellulose membrane at a rate of 1 µL/cm as the test (T) and control (C) lines, respectively. Subsequently, UCP-conjugating mAb conjugate (1 mg/mL, 30 µL/cm) was fixed in the glass fiber as the conjugate pad. The conjugate pad and membrane prepared by various candidate mAbs were paired; the optimized pair was selected for strip fabrication. The assembled strip was placed in the cartridge, with the sample-adding and result-scanning windows located above the sample pad and nitrocellulose membrane, respectively (Figure 1). During testing, the sample was initially diluted 10 times with the sample-treating buffer [0.03 M phosphate buffer (PB) that contains 1% BSA, 0.5% SDS, 0.1 M NaCl, and 0.01% NaN_3_, pH 7.2]. Exactly 100 µL of the diluted sample was applied to each strip. After 15 min, the strip was scanned with a UPT biosensor (UPT-3A) to obtain the results.

UPT-3A is similar to a previously described single-channel biosensor [30], which is equipped with a laser diode to emit 980 nm excited light and a photomultiplier tube to transform a 541.5 nm UCP-sourced emitted light to an electric signal (Figure 1). UPT-3A with an upgraded design was used to improve operational convenience. For the hardware, integrated keys were fabricated on the front panel to replace the mouse and keyboard. A platform was installed in the biosensor to settle the strips. When the “measure” key (all keys were in Chinese) was first pressed, the platform was stretched out, and the strips were settled on the platform. The button was pressed again, and the platform was drawn back and began to scan automatically. A radiofrequency identification device (RFID) was installed to automatically receive all information on a specific test from the built-in RFID chip of the UPT–LF strip (Figure 1). The information included the test item, cut-off threshold, quantitation parameters, and expiry date of the strip. For the software, a mathematical algorithm was designed to automatically fix the highest point and two inflection points of a signal peak. The integral value of that area was determined, and the value was used as the specific signal of the immunological reaction. Peak areas corresponding to the test and control lines were referred to as T and C values, respectively; the ratio of T/C was the result of the measurement [23], [35]. Samples with T/C ratios higher than the cutoff threshold (mean +3SD of T/C ratios corresponding to the blank) were considered positive and vice versa. Scanning and analysis were accomplished within 30 s by pressing a single key. To detect a series of samples, multiple strips were arranged in parallel, and the samples were added to the corresponding strip every 30 s. Each strip could be scanned following the addition of the sample for 15 min.

### Evaluation of sensitivity, linearity, and precision

PB was used as the blank sample and tested 10 times by each type of UPT–LF strip. Subsequently, the mean +3SD of T/C values were set as the cut-off thresholds that corresponded to the detected bacteria. Standard samples of *B. anthracis* spore, *B. melitensis* M55009, and *Y. pestis*, with concentrations ranging from 10^3^ cfu·mL^−1^ to 10^10^ cfu·mL^−1^, were prepared by serial dilution with PB. Each sample was detected in triplicate by the corresponding strip.

### Specificity evaluation

The specificity of the Ban-UPT–LF strip was determined using 12 bacterial strains [*B. atrophaeus* (two isolates) spore, *B. cereus* (three isolates) spore, *B. subtilis* (three isolates) spore, *B. thuringiensis* (two isolates) spore, *B. melitensis* M55009, and *Y. pestis*] with concentrations ranging from 10^5^ cfu·mL^−1^ to 10^9^ cfu·mL^−1^. Thirteen bacterial strains (*E. coli* O157:H7, *S. choleraesuis*, *S. enteritidis*, *S. paratyphi* A, *S. paratyphi* B, *S. paratyphi* C, *S. typhi*, *S. typhimurium*, *V. cholerae* O1, *V. cholerae* O139, *Y. enterocolitica*, *B. anthracis* spore, and *Y. pestis*), with concentrations ranging from10^6^ cfu·mL^−1^ to 10^9^ cfu·mL^−1^, were used to evaluate the specificity of the Bru-UPT–LF strip. Finally, the specificity of Ype-UPT–LF was determined by using 10 bacterial strains (*Y. aldovae*, *Y. enterocolitica*, *Y. intermedia*, *Y. kristensenii*, *Y. mollaretii*, *Y. pseudotuberculosis*, *Y. rohdei*, *Y. ruckeri*, *B. anthracis* spore, and *B. melitensis* M55009), with concentrations ranging from 10^4^ cfu·mL^−1^ to 10^9^ cfu·mL^−1^. Specific targets with the same concentrations were set as the positive controls.

### Evaluation of sample tolerance

#### Evaluation of single-factor theoretical tolerance

Immunological assays based on the capillarities of microporous materials cause the pH, ion strength, viscosity, and biological matrices (biomacromolecules) of the sample to noticeably interfere with the accuracy of the test. pH tolerance was determined by HCl and NaOH solutions, and ion strength tolerance was obtained from KCl–NaCl saline solutions. Glycerol and PEG20000 were selected as the representative micromolecules and macromolecules, respectively, to change the liquid viscosity. BSA and casein were selected as the representatives of serum-derived albumin and milk-derived phosphoproteins, respectively, to block immune reactions as biological matrices. All solutions were prepared with distilled water. Specific concentration points for interference reagents were determined according to the performance of each UPT–LF strip. *B. anthracis* spore, *B. melitensis* M55009, and *Y. pestis* were spiked into each solution of a specific reagent with a final concentration of 10^5^ cfu·mL^−1^ to 10^9^ cfu·mL^−1^, 10^5^ cfu·mL^−1^ to 10^9^ cfu·mL^−1^, and 10^4^ cfu·mL^−1^ to 10^8^ cfu·mL^−1^, respectively. Each simulated sample was tested by the corresponding UPT–LF strip in triplicate.

#### Evaluation of real sample tolerance

Various kinds of powder and viscera were selected as potential samples for anti-bioterrorism and zoonotic investigation. Seven kinds of powder, namely, flour, fruit juice, gourmet powder, milk powder, putty powder, soil, and sucrose, were combined with 0.03 mol·L^−1^ of PB to obtain solutions. Decomposed and fresh viscera of Balb/c mice were ground and homogenized in PB. The concentration of each solution was high and nearly close to the saturation point. *B. anthracis* spore, *B. melitensis* M55009, and *Y. pestis* were spiked into the aforementioned specimens at 10^5^ cfu·mL^−1^, 10^5^ cfu·mL^−1^ to 10^6^ cfu·mL^−1^, and 10^4^ cfu·mL^−1^, respectively. Each simulated sample was tested thrice using the corresponding UPT–LF strip.

### Evaluation of operation error tolerance

The standard operation was set as the control. Exactly 10 µL of the sample was mixed with 90 µL of the sample-treating buffer to obtain 100 µL of loading mixture, which was applied per strip. Each step of the liquid measurement procedure was analyzed to evaluate the operation error tolerance. To evaluate the tolerance of sample measure error, sample and sample-treating buffer were mixed at ratios of 5∶90, 20∶90, and 30∶90, and then 100 µL of the loading mixture was applied per strip. To evaluate the tolerance of the sample-treating buffer measure error, sample and sample-treating buffer were mixed at ratios of 10∶70, 10∶110, and 10∶130, and then 100 µL of the loading mixture was applied per strip. To evaluate the tolerance of the loading mixture measure error, sample and sample-treating buffer were mixed at standard ratios of 10∶90, and 70, 110, and 130 µL of the loading mixtures were applied per strip, respectively. *B. anthracis* spore (10^5^ cfu·mL^−1^), *B. melitensis* M55009 (10^6^ cfu·mL^−1^), and *Y. pestis* (10^4^ cfu·mL^−1^) were used as positive samples. Each sample was tested thrice using the corresponding UPT–LF strip.

## Results and Discussion

### Sensitivity, linearity, and precision of the UPT–LF assay

**Figure 2:**
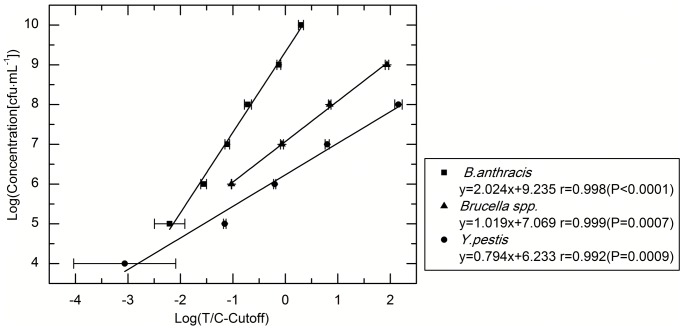
Sensitivity, linearity, and precision of the UPT–LF assay. For the detection of *B. anthracis* spore, *Brucella* spp., and *Y. pestis*, the sensitivity of the UPT–LF assay was 10^5^, 10^6^, and 10^4^ cfu·mL^−1^, respectively. The coefficients of variation (CV) of the three tests corresponding to each sample were all less than 15%.

Performance of the UPT–LF assay was initially evaluated using bacteria diluted in PB. The results gathered from the performance evaluation were used as standard in the evaluation of different interference factors. Sensitivity was set to the lowest concentration of dilution, with the T/C ratio higher than the cut-off threshold. For the detection of *B. anthracis* spore, *Brucella* spp., and *Y. pestis*, the sensitivity of the UPT–LF assay was 10^5^, 10^6^, and 10^4^ cfu·mL^−1^, respectively (Figure 2). The correlation coefficient (*r*) of the linear correlation analysis and the formula of the linear regression analysis, with the logarithm of T/C-cutoff as *x* and the logarithm of concentration as *y*, were used to evaluate linearity and achieve bacteria quantitation, respectively. The *r* of the linear correlation analysis was larger than 0.99, with linear quantitative values ranging from 10^5^ cfu·mL^−1^ to 10^10^ cfu·mL^−1^, 10^6^ cfu·mL^−1^ to 10^9^ cfu·mL^−1^, and 10^4^ cfu·mL^−1^ to 10^8^ cfu·mL^−1^ for *B. anthracis* spores, *Brucella* spp. and *Y. pestis*, respectively. The coefficients of the variation in the three tests corresponding to each sample were all less than 15%, guaranteeing the quantitation precision.

In this study, SDS was used as the surface-active agent in the sample-treating buffer to reduce the non-specific reaction by improving the hydrophilicity of biological macromolecules and also to facilitate the release of UCP particles from the conjugate pad. The covalent bonds between UCP particles and biological macromolecules are stable and free from the negative influence of SDS. The SDS concentration must be carefully optimized to reduce its potential negative influence. The bacteriolytic effect of SDS may also slightly improve the sensitivity of the test by increasing the amount of dissociative antigenic determinants.

### Specificity of the UPT–LF assay

**Figure 3:**
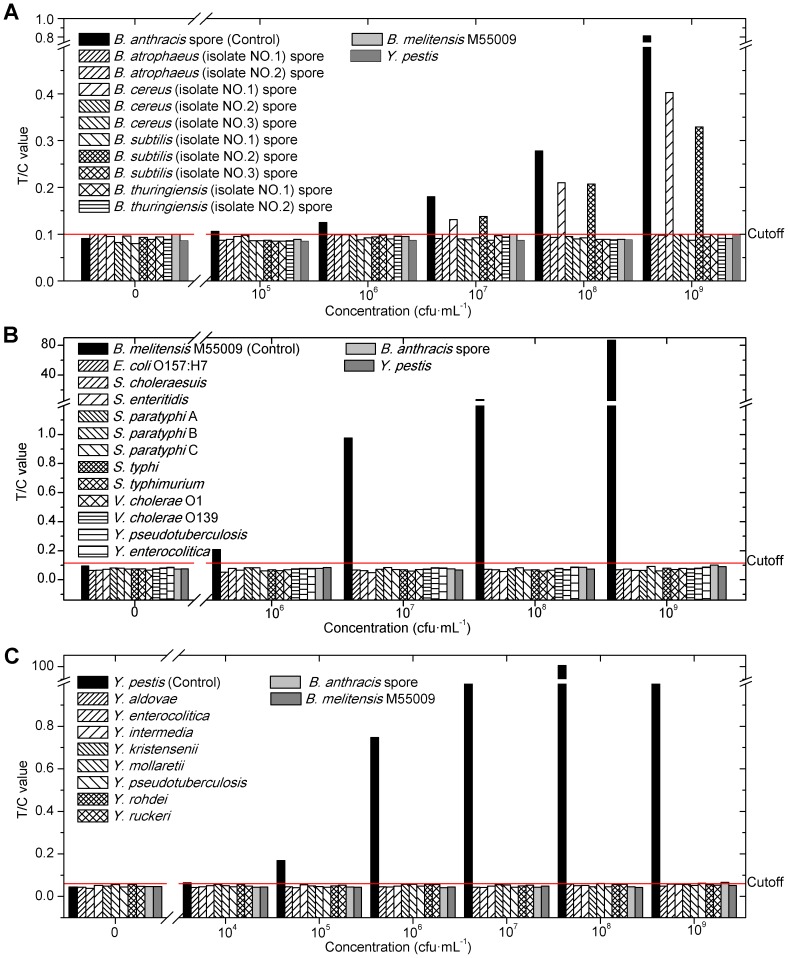
Specificity of the UPT–LF assay.

Bacteria that shared close genetic relatedness and similar transmission routes with the target were selected to evaluate the specificity of each kind of strip. Figures 3B and C show that the Bru-UPT-LF strip and the Ype-UPT-LF strip had high specificity and did not produce false-positive results even when the concentration of nonspecific bacteria was very high (10^9^ cfu·mL^−1^). However, the specificity of the Ban-UPT-LF strip was slightly disappointing (Figure 3A). Although significant differences were found between specific and nonspecific signals, *B. cereus* (isolate No. 1) and *B. subtilis* (isolate No. 2) spores began to cause a false-positive result at 10^7^ cfu·mL^−1^. No cross reaction occurred when the contaminating non-specific bacteria were at a lower concentration. The cross-reaction was caused by the monomorphic nature of *Bacillus* spp. that creates spore cell surface antigens [36] highly similar to those of some strains of *B. cereus*. Thus, the strains were even indistinguishable by PCR based on plasmids *pXO1* and *pXO2* encoding the toxin and capsule antigen, respectively [37].

### Sample tolerance of the UPT–LF assay

*B. anthracis* spore, *Brucella* spp., and *Y. pestis* are pathogens with dual identities, namely, zoonotic pathogens and biowarfare/bioterrorism agents. Given their dual identities, the targets of these species may exist in different samples (typically viscera and “white powder”) with various properties, which can be roughly classified into four: pH, ion strength, viscosity, and biological matrix (bio-macromolecule). UPT–LF assay is a microporous material-based immunological reaction. pH, ion strength, and biological matrix (blocking effect of bio-macromolecules) have important functions in the specificity and sensitivity of immunological reactions. Viscosity matters remarkably change the capillarity of microporous material. In this study, two experiments were carried out. The first experiment investigated the theoretical tolerance of the UPT–LF assay to single-factor interference. The second experiment determined the actual ability of the UPT–LF assay to detect real samples, including various viscera and powders. The results corresponding to the three kinds of strips were gathered to demonstrate the performance of the UPT–LF assay.

#### Theoretical tolerance to single-factor interference in the model samples

The serially diluted standard samples with PB were used as the controls for various interference factors. The T/C ratios of each strip under the interference of various factors with different concentrations (corresponding to the quantitative range) did not show significant change, except 0.1 mol·L^−1^ NaOH (pH 13), which altered the immunological reaction and led to a noticeable signal decrease (data not shown). This result indicates that the holistic signal response of the UPT–LF assay is relatively stable and promises accurate quantitation.

**Figure 4:**
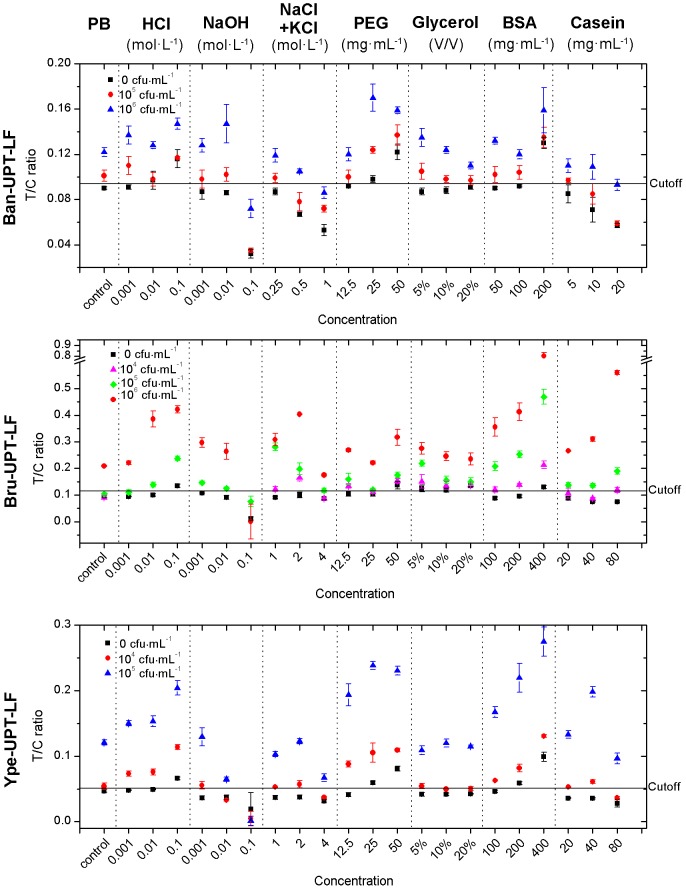
Tolerance of the UPT–LF strips to single-factor interference. The UPT–LF strip maintained sensitivity and specificity [T/C ratio of the positive sample (red circles) is higher than the cut-off threshold and T/C ratio of the negative sample (black squares) is lower than the cut-off threshold] and even improved the sensitivity at certain concentrations of the interference factors [T/C ratio of the positive sample (purple triangles, green diamonds) is higher than the cut-off threshold].

The T/C ratios of negative and weak positive samples (with concentration reaching the sensitivity) demonstrate the specificity and sensitivity of the test under the influence of interference factors (Figure 4). The highest concentration of an interference factor that does not decrease the specificity (i.e., no false-positive result for the negative sample test) and the sensitivity (i.e., no false-negative result for the weak positive sample test) of the test is defined as the tolerance limit. The tolerance limits of the three kinds of UPT–LF strips under the influence of various interference factors were determined (Table 1).

**Table 1:** Tolerance limits of UPT–LF assay to pH value, ion strength, viscosity, bio-macromolecule, and real samples.

Interference Factor		Unit	Ban-UPT–LF	Bru-UPT–LF	Ype-UPT–LF
pH value	HCl	mol·L^−1^	≤0.001 (pH 3)	≤0.01 (pH 2)**	≤0.01 (pH 2)
	NaOH	mol·L^−1^	≤0.01 (pH 12)	≤0.01 (pH 12)**	≤0.001 (pH 11)
Ion strength	NaCl+KCl	mol·L^−1^	≤0.25	≥4**	≤2
Viscosity	PEG20000	mg·mL^−1^	≤12.5	≤25**	≤12.5
	Glycerol	%(V/V)	≥20%	<5%	≤5%
Bio-macromolecule	BSA	mg·mL^−1^	≤100	≤200**	≤100
	Casein	mg·mL^−1^	≤5	≥80**	≤40
Power	Flour	mg·mL^−1^	≤100	≥200	≤50
	Fruit juice	mg·mL^−1^	≤100	≤50**	≤50
	Gourmet powder	mg·mL^−1^	≥400	≥400**	≤50
	Milk powder	mg·mL^−1^	≤25	≤200**	≥400
	Putty powder	mg·mL^−1^	≥200	≥200**	≤50
	Soil	mg·mL^−1^	≥400	≥400**	≥400
	Sucrose	mg·mL^−1^	≤100	≥400**	≥400
Viscera	Fresh heart	mg·mL^−1^	≥800	≥800	≥800
	Fresh liver	mg·mL^−1^	≤50	≤200**	≤50
	Fresh lung	mg·mL^−1^	≤400	≥800	≤100
	Fresh spleen	mg·mL^−1^	≤200	≥400	≤100
	Decomposed heart	mg·mL^−1^	≤100	≥400**	≤100
	Decomposed liver	mg·mL^−1^	≤50	≤100**	≤200
	Decomposed lung	mg·mL^−1^	≤100	≤200**	≤200
	Decomposed spleen	mg·mL^−1^	≤100	≤100**	≤100

** The sensitivity of UPT-LF strip improved at that tolerance limit.

The tolerance of the UPT–LF assay to pH ranged from 2 to 12, with extreme acid and basic backgrounds leading to false-positive and false-negative results, respectively. Ion strength showed a similar effect with NaOH solution. Saline solution with a high concentration resulted in the simultaneous decrease in the T/C ratios of negative and positive samples. This simultaneous decrease is a phenomenon that agrees well with the notion that ion strength can eliminate the nonspecific binding of immunological reaction at a proper concentration but damage the specific binding at an unsuitably high concentration. The maximum tolerated concentration (tolerance limit) of Ban-UPT-LF strip was 0.25 mol·L^−1^, whereas that of Bru-UPT-LF strip was more than 4 mol·L^−1^. In viscosity adjustment, the maximum tolerance limits of the UPT–LF assay to PEG20000 and glycerol were 25 mg·mL^−1^ and more than 20% (V/V), respectively. The macromolecule and the micromolecule exerted different influences on the assay. PEG20000 noticeably increased the signal, resulting in a false-positive result (i.e., destroyed the specificity of the test), whereas glycerol mainly decreased the signal. The tolerance limits of the three kinds of strip to PEG20000 were similar, but the tolerance of the three kinds of strip to glycerol was significantly different from that of Bru-UPT-LF, which was below 5%, and from that of Ban-UPT-LF, which was above 20%. As a rough representative of the bio-macromolecules in the biological matrix, the influence of BSA and casein was similar to that of PEG20000 and glycerol. The former simultaneously increased nonspecific and specific binding, whereas the latter decreased them. Therefore, specificity and sensitivity were damaged. The maximum tolerance limits to BSA and casein were 200 mg·mL^−1^ and more than 80 mg·mL^−1^, respectively.

Under the interference of various factors, the stability of the UCP optical characteristic and the UCP–biomolecule conjugate was critical. The properties of the antibodies in the UPT–LF strips were also important and especially noticeable in Bru-UPT-LF. The sensitivity and/or specificity of Ban-UPT-LF and Ype-UPT-LF were threatened by interference factors. The tolerance limits were determined as the highest concentration at which two kinds of strip could maintain the same specificity and sensitivity as the PB control. However, for Bru-UPT-LF, most interference factors (except glycerol) significantly improved the sensitivity. The sensitivity of Bru-UPT-LF changed from 10^6^ cfu·mL^−1^ to 10^4^ cfu·mL^−1^, but specificity was not damaged. Many reasons can account for this phenomenon. For the Bru-UPT-LF assay under the interference of NaOH, ion strength and casein, T/C ratios of the negative samples decreased, whereas those of the positive samples increased. However, the T/C ratios of both samples simultaneously decreased for Ban-UPT-LF and Ype-UPT-LF, thus generating false-negative results. For the Bru-UPT-LF assay under the interference of HCl, PEG20000, and BSA, T/C ratios of the positive samples increased, whereas those of the negative samples remained unchanged. However, the T/C ratios of both samples increased simultaneously for Ban-UPT-LF and Ype-UPT-LF, and false-positive results were generated.

#### Tolerance to real sample interference

**Figure 5:**
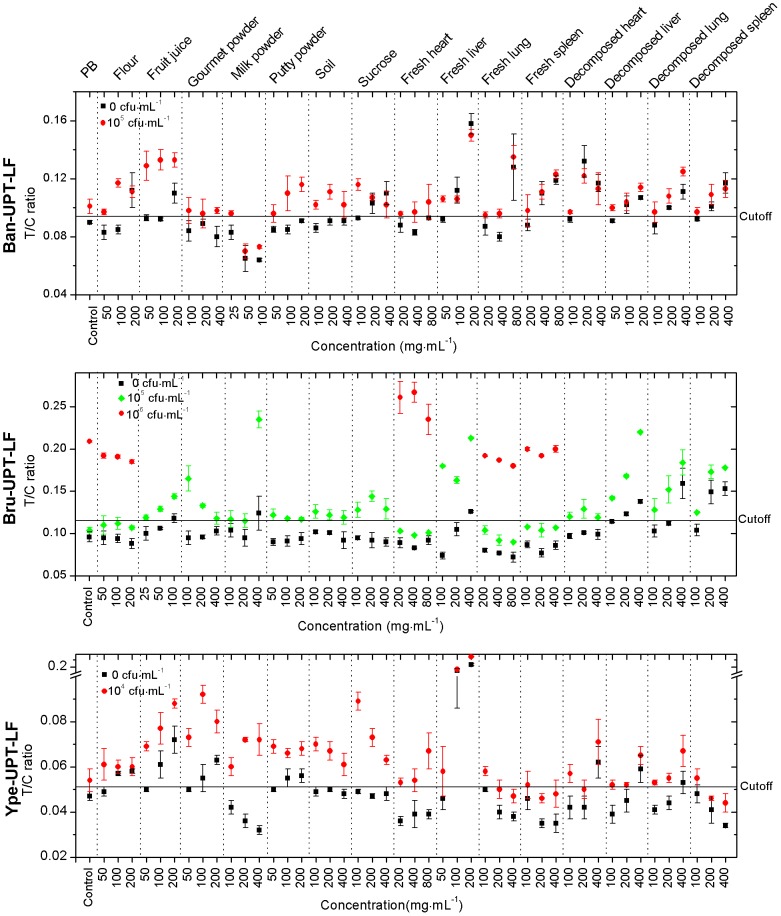
Tolerance of the UPT–LF strips to real sample interference. Under the influence of powders and viscera with gradient concentrations, UPT-LF strip maintained the sensitivity and specificity [T/C ratio of the positive sample (red circles) is higher than the cut-off threshold and T/C ratio of the negative sample (black squares) is lower than the cut-off threshold] and even improved the sensitivity at certain concentrations of the real samples [T/C ratio of the positive sample (green diamonds) is higher than the cut-off threshold].

The T/C ratios of the negative and weak positive samples under the influence of real samples are shown in detail in Figure 5. The properties of real samples as a complex of interference factors resulted in the complicated influence on UPT–LF strips fabricated by antibodies with various properties. Given the mild nature of gourmet powder, putty powder, soil, and sucrose as well as the close arrangement of the cardiac muscle cells of fresh heart that could not be easily combined with bacteria, the UPT–LF assay was not significantly affected by the five kinds of samples. Moreover, it showed ideal tolerance limits (reaching the highest interfering concentrations) (Table 1), except for the false-positive results of the Ban-UPT-LF strip for sucrose and those of the Ype-UPT-LF strip for gourmet powder and putty power at high interfering concentrations. By contrast, fruit juice with low pH, flour with high viscosity, fresh liver, decomposed heart, decomposed liver, decomposed lung, and decomposed spleen with thick and diversiform bio-macromolecules at high interfering concentrations caused false-positive results by increasing the signal of the negative and positive tests simultaneously and restricting the tolerance limit to a relatively lower level (Table 1). The only exceptions were the non-influence of flour and decomposed heart for Bru-UPT-LF strip and the false-negative of decomposed spleen for Ype-UPT-LF strip. The influences of milk powder, fresh lung, and fresh spleen on the UPT–LF assay were more complicated. At high interfering concentrations, fresh lung and fresh spleen samples led to false-positive results, no influence, and false-negative results for the Ban-UPT-LF, Bru-UPT-LF, and Ype-UPT-LF strips, respectively. Milk powder led to false-negative, false-positive, and no influence results for the Ban-UPT-LF, Bru-UPT-LF, and Ype-UPT-LF strips, respectively.

Similar to that in single-factor interference, the Bru-UPT-LF strip showed the strongest tolerance to real samples, with tolerance limits to flour, fresh heart, fresh lung, and fresh spleen reaching the highest interfering concentrations (Table 1). Aside from the abovementioned real samples, the remaining samples significantly increased the sensitivity of Bru-UPT-LF strip from 10^6^ cfu·mL^−1^ to 10^5^ cfu·mL^−1^ without any changes in specificity (Figure 5).

### Operation error tolerance of the UPT–LF assay

**Figure 6:**
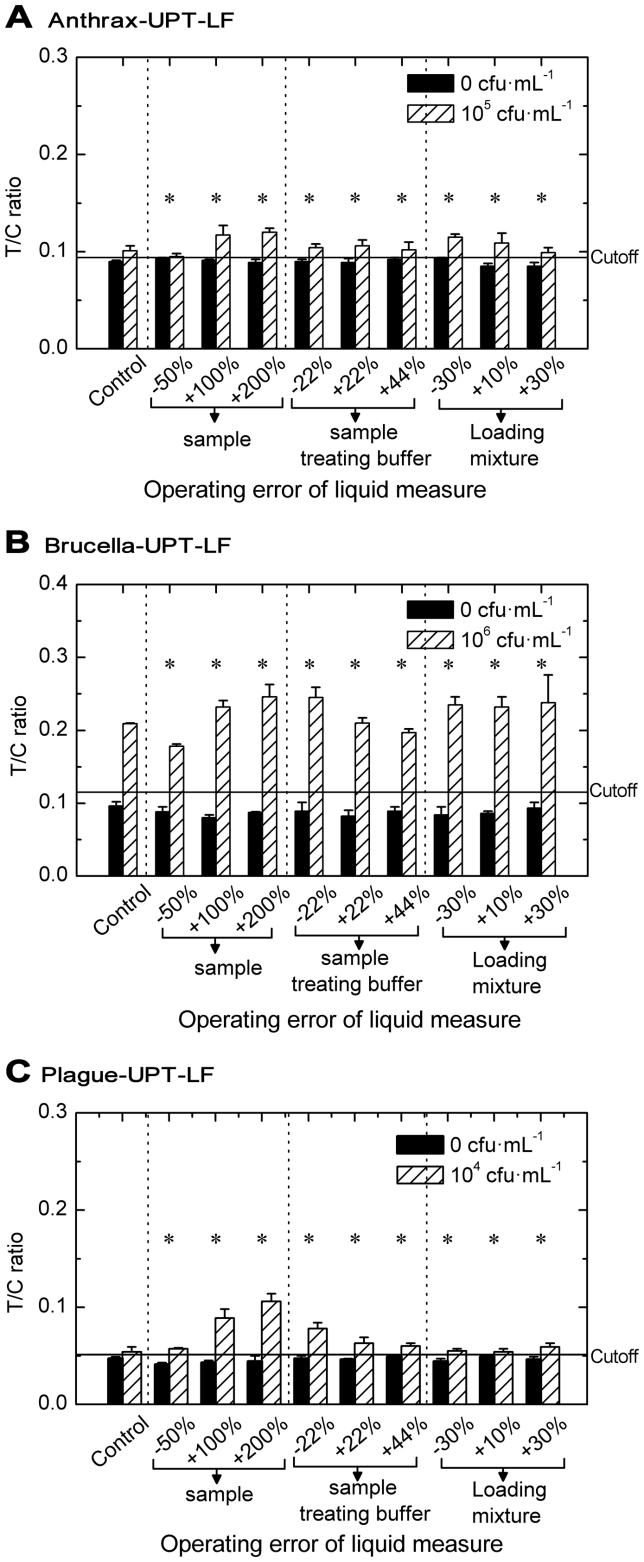
Tolerance of the UPT–LF assay to operation error. Ban-UPT-LF (A), Bru-UPT-LF (B), and Ype-UPT-LF (C) maintained their sensitivity and specificity (*) under the operation error of samples measure (from −50% to +200%), sample treating-buffer measure (from −22% to +44%), and loading mixture measure (from −30% to +30%).

As POCT assay is operated by non-professionals for level-one emergency response, operation error caused by apparatus and operator error cannot be avoided. Therefore, the influence of operation error on sensitivity and specificity should be evaluated. For the UPT–LF assay, the possible operation errors came from the sample measure, sample-treating buffer measure, and loading mixture measure. In Figure 6, with the results of the standard operation used as control, the T/C ratios of negative and weak positive samples under the influence of operation error are shown in a bar graph to indicate the changes in specificity and sensitivity. The volume deviation of the sample (from −5% to +200%), sample-treating buffer (from −22% to +44%), and loading mixture (from −30% to +30%) did not cause any loss of sensitivity and specificity to the UPT–LF assay. The volume change in sample and sample-treating buffer altered the concentration of detected targets in the loading mixture and led to a corresponding decrease or increase in T/C ratios. However, when the volume of the sample and the sample-treating buffer changed with the same proportion, the signals were mainly dependent on the effective volume of loading mixture flowing through the detection line, which was limited by the flow rate of the nitrocellulose filter and the bed volume of absorbent pad. Thus, no significant change in T/C ratios was observed for the error of the loading mixture measure.

## Conclusions

The concentration of bacteria is often high in infected animals from the nature foci and in the powders used in bioterrorism. Therefore, a rapid screening test is required for routine disease surveillance and incident management. Pre-treatment or bacterial culture of the sample, which is needed for the detection of complex specimens, always takes a long time and entails a complicated operation process that cannot be performed by a non-professional. An improved UPT–LF assay applied for POCT was developed and evaluated comprehensively in this study. Sample tolerance and operation error tolerance were analyzed to reveal the feasibility and practical application of the newly developed technology. A complex sample could be directly detected through simple dissolution or homogenization by a non-professional. The stable optical characteristics of UCP and the covalent bond between UCP and biomolecules ensure that the UPT-LF assay is free from sample influences and non-standard operation errors. Detection using a biosensor can be realized by only pressing a key on the front panel of an upgraded UPT biosensor. Indeed, the procedure is simple and convenient for non-professional operators.

In the LF assay, some specific common antigens, which are surface exposed and important for virulence, are used in the preparation of antibodies that can recognize as many species as possible, such as F1 antigen [22] and plasminogen activator protein [16] of *Y. pestis*, anthrose tetrasaccharide of *B. anthacis* [19], outer membrane proteins and O-polysaccharide of *Brucella* [20], [21], [38]. However, most surface antigens are difficult to isolate and purify because they are amphipathic molecules. Even antigens obtained by bioengineering technology tend to lose their activity. To ensure the activity, specificity and recognition of as many species as possible, we used the representative inactivated bacteria to immunize the Balb/c mice prior to antibody preparation. The preliminary mAb was chosen by high-throughput screening using ELISA method coated by the representative bacteria. Specificity was also evaluated using the bacteria that shared close genetic relatedness and similar transmission routes with the target. The results demonstrated that UPT–LF assay with antibodies prepared by the methods had high specificity and yielded no false-positive results even when the concentration of nonspecific bacteria reached 10^9^ cfu·mL^−1^.

After comprehensive evaluation, we believe that the UPT–LF assay can be used as an alternative method for infectious disease surveillance in the natural foci, point-of-care screening, environmental surety of health, and incident management of biowarfare and bioterrorist attacks.
